# CHILDREN’S HEALTH: Less Pollution, Less Earache?

**DOI:** 10.1289/ehp.117-a540a

**Published:** 2009-12

**Authors:** Carol Potera

**Affiliations:** **Carol Potera**, based in Montana, has written for *EHP* since 1996. She also writes for *Microbe*, *Genetic Engineering News*, and the *American Journal of Nursing*

Children exposed to secondhand cigarette smoke suffer more ear infections. Irritating toxicants in cigarette smoke, such as carbon monoxide (CO) and sulfur dioxide (SO_2_), also are constituents of vehicular emissions, so it’s not surprising that children exposed to high levels of traffic pollution also have more ear infections, as shown by studies published in the September 2006 *EHP* and the May 2007 *European Respiratory Journal*. In the newest twist on the air pollution/ear infection connection, researchers have linked improvement in U.S. air quality over a decade with a reduction in the prevalence of pediatric ear infections.

Otolaryngologists Nina Shapiro of the University of California, Los Angeles, and Neil Bhattacharyya of Harvard University obtained data for 126,060 children, mean age 8.6 years, collected as part of the National Health Interview Survey between 1997 and 2006. Shapiro and Bhattacharyya identified cases of frequent otitis media (defined in the survey as three or more ear infections in the previous 12 months), respiratory allergy, and seizures. Seizures were included as a control condition believed to be unrelated to air pollution. Data on concentrations of CO, SO_2_, nitrogen dioxide (NO_2_), and particulate matter (PM) for the same 10-year period came from the U.S. Environmental Protection Agency (EPA). Elevated levels of all four pollutants are strongly associated with deficits in respiratory health.

During the study period, air quality steadily improved, and the incidence of recurrent ear infections fell, but the incidence of allergy or seizures did not change. SO_2_ and NO_2_ were more strongly associated with frequent otitis media than were CO and PM. The researchers reported the results at the American Academy of Otolarynology—Head and Neck Surgery Foundation national meeting in October 2009. Bhattacharyya elaborates, in figures not presented at the meeting, that in 1997 there were about 5.8 million cases of children with frequent otitis media, whereas by 2006, the number of frequent otitis media cases had fallen to 4.1 million.

These preliminary results suggest it may be possible to “track the effects of environmental pollution on one disease, in this case ear infections, and see if children benefit from a greener Earth,” says Udayan Shah, co-director of fellow and resident education in pediatric otolaryngology at Nemours–Al DuPont Hospital for Children in Wilmington, Delaware. The findings, while intriguing, will need to be confirmed in studies of individual children.

Exactly how air pollutants might contribute to otitis media remains unknown. However, all four toxicants are known to cause inflammation that restricts the movement of respiratory cilia that clear toxicants. “The lining of the middle ear is similar to the respiratory tract mucosa,” notes Shapiro, suggesting that similar mechanisms may be involved. In a study published in the April 1989 (part 1) issue of the *Annals of Otology, Rhinology, and Laryngology*, Y. Ohashi and colleagues found that SO_2_ depressed cilia function in guinea pigs’ ears.

The Clean Air Act revisions of 1990 strengthened the EPA’s enforcement of stringent regulations aimed at improving air quality to benefit the nation’s health, with the added benefit of reducing medical costs. In the February 2004 issue of *EHP*, Eva Y. Wong and colleagues estimated that reductions in air pollution by 2010 as a result of the Clean Air Act could save up to $2 billion in children’s respiratory health costs alone. If the current findings bear out, the savings could be substantial for otitis media costs, which may exceed $5 billion annually, according to a report in the June 2000 issue of *Pediatrics*.

In future projects, Shapiro and Bhattacharyya will explore how changes in average annual temperature relate to respiratory illnesses in adults and children. In the first study of this type, Bhattacharyya found a statistically significant association between increased prevalence of sinusitis and increased annual temperatures between 1998 and 2006, as described in the October 2009 issue of *The Laryngoscope*.

